# Evolution of BRET Biosensors from Live Cell to Tissue-Scale *In vivo* Imaging

**DOI:** 10.3389/fendo.2013.00131

**Published:** 2013-09-23

**Authors:** Abhijit De, Akshi Jasani, Rohit Arora, Sanjiv S. Gambhir

**Affiliations:** ^1^Molecular Functional Imaging Laboratory, ACTREC, Tata Memorial Centre, Navi Mumbai, India; ^2^MIPS, Department of Radiology, School of Medicine, Stanford University, Stanford, CA, USA

**Keywords:** bioluminescence resonance energy transfer, luciferase, fluorescent proteins, optical imaging, protein–protein interactions, cell-based assay

## Abstract

Development of bioluminescence resonance energy transfer (BRET) based genetic sensors for sensing biological functions such as protein–protein interactions (PPIs) *in vivo* has a special value in measuring such dynamic events at their native environment. Since its inception in the late nineties, BRET related research has gained significant momentum in terms of adding versatility to the assay format and wider applicability where it has been suitably used. Beyond the scope of quantitative measurement of PPIs and protein dimerization, molecular imaging applications based on BRET assays have broadened its scope for screening pharmacologically important compounds by *in vivo* imaging as well. In this mini-review we focus on an in-depth analysis of engineered BRET systems developed and their successful application to cell-based assays as well as *in vivo* non-invasive imaging in live subjects.

## Introduction

In the post-genomic era, rapid functional evaluation of protein–protein interactions (PPIs), protein phosphorylation, or protease function, which play a key role in various cellular processes such as signal transduction, cell division, transport, etc., in live cell condition is essential. Moreover, the study of such PPIs in normal and diseased cells can help shed light in the understanding of the diseases and to develop suitable therapies. For a long time, conventional biochemical assays like co-immunoprecipitation (1, 2), gel-filtration chromatography (3), sandwich enzyme-linked immunosorbent assay (ELISA) (4), etc., have been used in the investigation of PPIs. These assays though successful, do not suffice as imaging probes because they: (i) are essentially endpoint measurements, (ii) fail to provide spatio-temporal information on specific PPIs, (iii) require mechanical, chaotropic, or detergent based cell lyses, which may alter native PPIs in some cases (5, 6), (iv) are insensitive to transient interactions that regulate certain cellular processes, and (v) have little or no utility for *in vivo* imaging in live subjects. To overcome these limitations, non-invasive imaging approaches such as bioluminescence resonance energy transfer (BRET) have been developed over the last decade, which allow the study of PPIs in their native environment and are capable of providing a unified platform that can be translated from cell culture-based assays to the imaging of live subjects (6, 7). In this mini-review, we will be exploring some hitherto unexplained factors affecting the spectral pattern of several BRET systems and their successful application to cell-based assays as well as *in vivo* imaging of live subjects.

## Biophysical Basis of BRET

Bioluminescence resonance energy transfer is an intrinsic phenomena occurring in the organisms *Renilla reniformis* and *Aequorea victoria*. Exploiting the underlying principles of BRET from nature, literatures demonstrating BRET biosensor applications started since the year 1999. The BRET phenomenon that follows the Förster resonance energy transfer (RET) principle (8), occurs between two proximally situated chromophores – a bioluminescent donor such as a luciferase protein and a fluorescent protein (FP) acceptor with overlapping emission and excitation spectra respectively. Following donor excitation upon substrate addition, part of the electronic excitation energy of the donor is dissipated due to random collisions with other molecules while the remaining electronic relaxation energy is transferred to the acceptor molecule through non-radiative dipole–dipole coupling. Upon excitation, the acceptor molecule now emits its photonic energy at its characteristic wavelength. This results in a decrease in donor emission paralleled by an increase in acceptor emission. The strict dependence of BRET on the inter-chromophoric distance (1–10 nm) makes it an appropriate “molecular yardstick” for determining PPIs. This is true, since the average protein radius is ∼5 nm, which means that a positive BRET signal will only be detected if the two proteins come within ∼10 nm of each other, a distance that is an indicator of direct interaction between the two proteins (9). However, absence of a BRET signal does not necessarily mean that the two target proteins do not interact with each other. Lack of a signal can be accounted for by an unfavorable orientation between the donor and acceptor dipoles. The BRET ratios can be calculated as per Eqs. 1 and 2 (10).

(1) $$\mathrm{BRET}=\frac{BL_{\mathrm{emission}}\left(\mathrm{Acceptor}\;\lambda \right)-C_{f}\;\times \;BL_{\mathrm{emission}}\left(\mathrm{Donor}\;\lambda \right)}{BL_{\mathrm{emission}}\left(\mathrm{Donor}\;\lambda \right)}$$

where,

(2) $$C_{f}=\frac{BL_{\mathrm{emission}}{\left(\mathrm{Acceptor}\;\lambda \right)}_{\mathrm{donor}\;\mathrm{only}}}{BL_{\mathrm{emission}}{\left(\mathrm{Donor}\;\lambda \right)}_{\mathrm{donor}\;\mathrm{only}}}$$

In the above equation, BL_emission_ is the average radiance measured at the donor (Donor λ) or acceptor (Acceptor λ) filters in BRET-transfected or only donor transfected cells; the correction factor (*C*_f_) represents the BRET signal detected from cells transfected only with the donor plasmid. Upon subtracting this factor from the overall BRET ratio, one can get an idea of the dynamic range for a particular BRET pair. Moreover, since BRET-based assays are ratiometric, any variability due to assay volume or cell number variation or time point of measurement is nullified.

Until recently, the field of BRET-based biosensors has predominantly utilized two basic BRET systems, viz., BRET^1^ and BRET^2^. Developed by Xu et al. the BRET^1^ system combines *Renilla* luciferase (RLuc) with enhanced yellow fluorescent protein (EYFP) (11). However, the spectral resolution (separation of peak donor and acceptor emission spectra) achieved in BRET^1^ is ∼50 nm only, which is considered suboptimal for macroscopic imaging (12, 13). Another BRET system, named as BRET^2^, combining RLuc with a UV-excitable GFP variant viz., GFP^2^ (14, 15) was developed, that uses a coelenterazine analog-DeepBlueC™ (also known as coelenterazine 400a or Clz400) substrate, which shifts the emission maximum $\left(E_{m_{\max}}\right)$ of RLuc to 400 nm. GFP^2^ excites at a maximum $\left(E_{x_{\max}}\right)$ of 396 nm and emits photons at 510 nm. This yields a much larger spectral resolution of 110 nm and has enabled us to perform tissue-scale imaging using wideband filters for the first time (16, 17). However, successful tissue imaging with higher sensitivity of cells located deep inside the animal body calls for the design and development of BRET systems with more red-shifted emissions. This is because, at wavelengths below 600 nm, particularly in the blue-green regions of light, pigments like myoglobin and hemoglobin absorb a significant fraction of the visible light (18).

## Expansion of BRET Assay Formats

**Figure 1:**
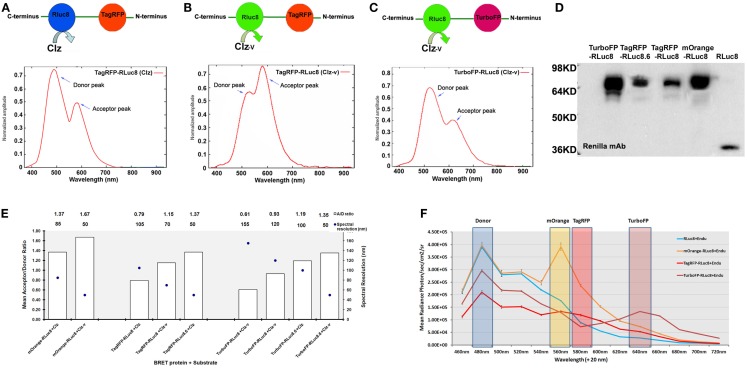
**Various new generation of BRET pairs reported using mutant *Renilla* luciferase proteins**. **(A)** Represents TagRFP-RLuc8 pair using normal coelenterazine (Clz) substrate. The bottom chart represents normalized amplitude vs. wavelength measured from the purified fusion protein added with Clz substrate. **(B)** Represents the same pair using the coelenterazine-*v* (Clz-*v*) substrate analog. **(C)** Represents TurboFP-RLuc8 pair using the Clz-*v* substrate. The chart represents normalized amplitude vs. wavelength measured from purified protein with Clz-*v* substrate. Note that in majority cases [e.g., **(A)** or **(C)**], typical normalized amplitude emission of the donor is higher than the acceptor emission output, whereas in some cases [e.g., **(B)**], due to efficient transfer of energy, normalized amplitude of acceptor emission is higher than the donor emission. **(D)** Represents western blot analysis of various BRET fusion proteins made by combining mutant *Renilla* luciferases (such as RLuc8 and RLuc8.6) with mOrange, TagRFP, and TurboFP fluorescence acceptors. As control, RLuc8 (RL8) protein of 35 kDa size was also shown on the right most lane. **(E)** BRET ratios (denoted as A/D ratio on *Y*-axis) are calculated by measuring the donor and acceptor $E_{m_{\max}}$ from each of these fusions in the presence of indicated Clz (or its analog) substrate from mammalian cells expressing the proteins. Spectral resolutions (difference between the donor and acceptor emission peaks) are also plotted on the *Z*-axis (as dark circles). One can observe an inverse relationship between the BRET ratio measured and the spectral resolution of the BRET partners which is more or less linear in fashion. In some cases, the BRET ratio was seen to be >1 which is theoretically not possible. This is because the values mentioned here are the raw measured values without accounting for the *C*_f_ value. Once the *C*_f_ value is added into the equation, the BRET ratios drop below 1. Note that either Clz-*h* or Clz-*v* substrate analogs can be used against the same fusion protein to fine tune the spectral resolution. **(F)** Spectral profile measured from live mammalian cells over-expressing RLuc8, mOrange-RLuc8, TagRFP-RLuc8, and TurboFP-RLuc8 fusion proteins. Cells over-expressing these proteins were exposed to live cell substrate EnduRen^TM^ (Endu) and emission profiles were imaged using IVIS spectrum imaging system loaded with 20 nm band-pass filters between 460 and 720 nm. This arrangement provides a multiplexing opportunity, where the same donor protein can be combined with multiple acceptors, whose $E_{m_{\max}}$ can be resolved by band-pass filter sets as indicated on the chart.

In the past few years, improvisations in various components of BRET such as luciferases, FPs, substrates, and instrumentations have contributed to the remarkable expansion in the range of BRET platforms available. Armed with these BRET vectors, the progress of molecular imaging to live cells, animals, and plants with varied applications has been made possible. With the advent of engineered RLuc variants with an elevated photon output and/or a red-shifted $E_{m_{\max}}$, *viz*., RLuc8 ($E_{m_{\max}}\;480\;\mathrm{nm;}$ four fold increase in photon output compared to RLuc) (19) and RLuc8.6 ($E_{m_{\max}}\;535\;\mathrm{nm;}$ ∼6-fold increase in photon output compared to RLuc) (20), new BRET systems in combination with FPs in the orange and red regions of emission spectra were developed (Figures 1A–D). Theoretically, the amplitude of donor emission should always exceed the acceptor emission (Figures 1A,C). However, we noted that in the spectral profiles of some of these newly developed BRET systems, the normalized amplitude at the donor emission was lower in comparison to that at the acceptor emission (Figure 1B). For example, in the case of TagRFP-RLuc8, only when Clz-*v* substrate was used (shifting the peak donor emission to 515 nm), the amplitude of TagRFP at 585 nm surpassed RLuc8 emission. To explain this anomaly, a deeper understanding of the RET principle is required. RET efficiency is essentially an interplay between the spectral overlap integral of the donor emission and acceptor excitation spectra, in addition to the quantum yield of the donor. We speculate that a donor bleed through signal coupled with the high degree of spectral overlap between RLuc8 and TagRFP (upon the use of Clz-*v*) that favors maximum energy transfer between the pair is detected at the acceptor filter, giving an unnaturally high peak. On a different note, if one tries to define the ideal BRET pair for tissue-scale imaging, it would be the one that gives a high spectral resolution with minimally compromising the BRET ratio. Based on the data compiled from the BRET systems available with us (Figure 1E), TurboFP and RLuc8.6-Clz combination would be the ideal BRET partners for both *in vitro* and *in vivo* imaging as they have a high BRET ratio (∼1.19) with an equally high spectral separation of 100 nm.

## BRET Options for Studying the Kinetics of Protein Interactions

A landmark development in the recruitment of BRET-based systems to capture the PPI kinetics was the creation of protected Clz analogs. The problem associated with the use of normal Clz substrates in live cells was their auto-oxidation, resulting in the loss of peak signal within 2–3 min, which further drops to ∼50% within 17 min. Consequently the measurement of long-term PPI kinetics is impossible using such substrates. To eliminate this problem, Levi et al. (21) reported chemical modifications to protect the putative oxygenation sites of Clz400 and demonstrated that depending on the protective modifications, long-term BRET^2^ monitoring was achievable. Similarly, another commercial source also developed EnduRen™ substrate that can be used specifically for live cell imaging (22). This is a protected form of coelenterazine-*h* with their active sites blocked by esters or oxymethyl ethers that are only released upon cleavage by intracellular hydrolytic enzymes. The absence of active Clz-*h* in the media significantly reduces the signal attenuation due to background auto-oxidation and the half-life of Clz-*h* increases. Moreover, a steady-state bioluminescence emission of EnduRen™ till about 24 h potentiates its use for monitoring dynamic changes in PPIs from live cell conditions.

Another commonly used luciferase is the North American Firefly luciferase (FLuc; $E_{m_{\max}}\;562\;\mathrm{nm}$) (23). A codon-optimized version of FLuc has been created by commercial sources for use in mammalian cells. The relatively slower and stable emission kinetics of its substrate, d-luciferin, makes it naturally suitable for kinetic measurements from live environments, obviating the need for any chemical modifications to its structure (24). FLuc has been reported to be used in a BRET system in conjunction with red FPs like DsRed (25) as well as with non-protein fluorophores such as Cy3 and Cy3.5 (26). However, its bulky size of 61 kDa, an obligate dependence on Mg^2+^ and ATP as its cofactors (27, 28) and finally, a low spectral resolution with the BRET partners reported so far, makes it a poor choice for BRET.

## Multiplexed BRET Options for Co-Lateral Interaction Studies

In addition to the simple PPIs assays, one might be interested to monitor two concurrent dependent/independent PPI events within the same cell. BRET multiplexing was employed in one of the GPCR studies to monitor the ubiquitination kinetics and its involvement in receptor regulation. Exploiting the distinct spectral emission properties of the RLuc substrates-Clz-*h* $(E_{m_{\max}}\;480\;\mathrm{nm)}$ and Clz400 $\left(E_{m_{\max}}\;400\;\mathrm{nm}\right)$, Perroy et al. co-expressed RLuc-β-arrestin and GFP^2^-ubiquitin along with a YFP-labeled vasopressin receptor (V_2_R-YFP) (29). In this way, depending on the substrate (Clz-*h* or Clz400) oxidized by RLuc, either BRET^1^ or BRET^2^ kinetics can be respectively detected. Appropriate negative controls, for instance, the use of Clz-*h* to detect negligible BRET transfer between RLuc and GFP^2^ can validate the authenticity of such experiments. Moreover, with the series of BRET systems that are now available to us, one can recruit either a single/dual luciferase system such as RLuc8 and RLuc8.6 with appropriate acceptor FPs (Figure 1F), which can facilitate BRET multiplexing of three to four candidate proteins. The ease with which this objective can be achieved and the requirement of only a single substrate, makes it a highly attractive option for co-lateral protein interaction studies.

## Multiplexed BRET Options for Studying Multi-Protein Complex

While dual-BRET techniques facilitate the concomitant monitoring of two different PPIs events, elegant approaches such as sequential RET (SRET) (30, 31), bimolecular-fluorescence complementation-BRET (BiFC-BRET) (32, 33), complemented donor-acceptor-RET (CODA-RET) (34), and bimolecular luminescence complementation-BiFC (BiLC-BiFC) (35, 36) have enabled the detection of interactions between higher order protein complexes. In the SRET technique, the three candidate proteins are fused to either RLuc donor or one of the two FP acceptors. In such a situation, a BRET process excites the first fluorescent acceptor, which will now serve as a fluorescence resonance energy transfer (FRET) donor for the second fluorescent acceptor. Two such systems, SRET^1^ (RLuc-YFP-DsRed) and SRET^2^ (RLuc-GFP^2^-YFP) utilizing Clz-*h* and Clz400 substrates respectively, were reported in literature, that could detect the heterotrimerization of cannabinoid CB_1_ receptor (CB_1_R), dopamine D_2_ receptor (D_2_R), and adenosine A_2A_ receptor (A_2A_R) as well as the assembly of G-protein subunits in living cells. Further, Navarro et al. successfully demonstrated the oligomerization of Calmodulin (CaM), A_2A_R, and D_2_R using SRET^2^ in live cells, which can open avenues for screening of potential drugs that specifically target these receptor interactions. In another study, the CB_1_R-D_2_R-A_2A_R interactions were studied using a BiFC (using N- and C-termini truncated forms of YFP) coupled with a luciferase protein to form a functional BRET system (BiFC-BRET). In yet another recent literature, a split luciferase complementation reconstituting the donor bioluminescence was paired to an acceptor FP to detect the BRET signal. This technique, termed CODA-RET can be used in the study of receptor oligomerization in presence of agonists/antagonists as well as in drug screening. Amalgamating the above two techniques, one can also employ both complemented donor along with bimolecuar fluorescence complementation (BiLC-BiFC) to form a functional BRET system that can explore the interaction of up to four proteins.

## BRET for Tissue-Scale Imaging

While we have progressed so far in terms of optimizing various BRET platforms with the aim to image PPIs non-invasively in their natural physiological environments *in vivo*, it has not yet been achieved completely. However, scientific endeavors have not been futile. With the introduction of the intensely cooled charge coupled device (CCD) camera-based optical imaging instrumentation, the ability to detect very dim photon signals from live cells in culture or from animal or plant tissues has become possible. To detect signals with detectors placed outside the animal subjects, the cells of interest present at a depth within the subject must produce sufficient signal. Here, primarily the use of red and NIR light signals is favored as they have lesser tissue attenuation and thus, better penetration capacity. Therefore, overall modification of existing assays to adapt them for non-invasive monitoring is a challenging task. Approaching the development of a single format imaging assay that can serve to measure PPIs from isolated single cells as well as physiologically relevant animal/plant models, both BRET^1^ and BRET^2^ strategies display some form of confinements. Therefore, while attempting live animal BRET assays, we have conducted serial experiments to identify an optimal BRET assay showing satisfactory performance as a single format assay (12, 16, 17). By now, we have introduced an ample variety of the red light emitting BRET vectors, many of which undoubtedly show superior performance over the previous assays used. By withdrawing the traditional method of BRET measurement using a microplate reader, we adapted a method for spectral separation of donor and acceptor signal by using black-box cooled CCD camera macro-imager (16). An important parameter to successfully adapt this imaging method was the use of the BRET formats with relatively large spectral resolution, which allows the selection of wide band-pass emission filters in the device. Thus the CCD camera-based macro-imaging instrument can measure BRET signals from lysed or live cells placed in multi-well plates. The same instrument can then be used for BRET measurement from whole organisms as well. A point worth noting here is that, BRET imaging from animal tissues is further complicated by the consideration of tissue attenuation factor. To address this, a double ratio (DR) which provides a depth-independent measure of the BRET signal in animal experiments was defined (Eq. 3) (7).

(3) $$\begin{array}{llll} \mathrm{DR} & =\frac{BL_{\mathrm{emission}}{\left(\mathrm{Acceptor}\;\lambda \right)}_{\mathrm{BRET}}\times \mu_{t}\left(\mathrm{Acceptor}\;\lambda \right)}{BL_{\mathrm{emission}}{\left(\mathrm{Donor}\;\lambda \right)}_{\mathrm{BRET}}\times \mu_{t}\left(\mathrm{Donor}\;\lambda \right)}∕\; & & \;\\ & \;\frac{BL_{\mathrm{emission}}{\left(\mathrm{Acceptor}\;\lambda \right)}_{\mathrm{donoronly}}\times \mu_{t}\left(\mathrm{Acceptor}\;\lambda \right)}{BL_{\mathrm{emission}}{\left(\mathrm{Donor}\;\lambda \right)}_{\mathrm{donoronly}}\times \mu_{t}\left(\mathrm{Donor}\;\lambda \right)} \end{array}$$

where, μ*_t_* denotes the total attenuation coefficient.

**Figure 2:**
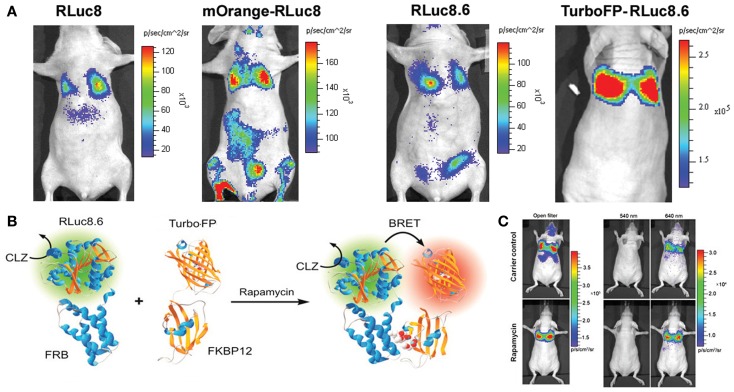
**Bioluminescence resonance energy transfer performance in deep tissue imaging experiments**. **(A)** Upper panel represents mouse images comparing improvements in the signal output from lungs. Mammalian cells engineered for equivalent over-expression of donor alone (RLuc8 or RLuc8.6) or BRET proteins (mOrange-RLuc8 or TurboFP-RLuc8.6) as marked, were compared. Note the photon output values in the reference color scale bars. Highest signal output from same number of cells placed within lungs was noted with TurboFP-RLuc8.6 BRET protein imaged with Clz substrate. **(B)** Schematic illustration of the most successful BRET format tested for monitoring the rapamycin induced FRB-FKBP12 association. **(C)** Representative bioluminescence images of nude mice with accumulated mammalian cells in the lungs which stably over-express FRB and FKBP12 interacting partners fused to RLuc8.6 and TurboFP respectively. Cells (3 × 10^6^ in 150 μL PBS) were injected through the tail vein, resulting in significant trapping in the lungs. One group of mice (*n* = 8) was injected 2 h before cell injection with 40 μg rapamycin dissolved in 20 μL DMSO and further diluted in 130 μL PBS administered through the tail vein. A second group of mice (*n* = 8) was injected with DMSO (20 in 130 μL PBS). Two hours after cell injection, the mice were injected i.v. with Clz substrate and sequentially imaged using open/donor/acceptor filters. Substrate-only control mice (*n* = 4) were used for background subtraction. The figure is partially represented with permission from PNAS (7).

The main bottleneck of extending FRET strategy in small animal evaluation is associated with the auto-fluorescence correction method. As light travels in and out from animal tissues, the resulting photon attenuation complicates the FRET ratio calculations. In this context, the exclusion of an external photon input makes BRET-based technologies more acquiescent for macro-scale imaging of PPIs. As represented in Figure 2, we have also done proof of principle studies by confirming the detection of the rapamycin-dependent interaction of FKBP12 and FRB from living animals (7, 12, 17). Following the successful BRET imaging from small animal model, macro-imaging of plant tissues was also reported (37). Using a modified electron bombardment-CCD camera coupled with a dual-view image splitter, visualization of the constitutive photomorphogenesis 1 protein (COP1) homo-dimerization using RLuc-EYFP BRET assay was demonstrated in the rootlet and cotyledons of tobacco seedlings in order to understand its repressive activity on light regulated development in plants. The same group had previously reported the use of a similar BRET assay in onion epidermal cells as well as in the *Arabidopsis* seedlings to study the effect of COP1 dimerization and its nuclear exclusion on the functional activity of COP1 (38). BRET is better adapted to plant imaging, since it circumvents the issues of photobleaching and auto-fluorescence of photosynthetic pigments as seen in the case of FRET. Considering careful validation of the PPIs in systematic, large-scale models using individual test cases, the molecular imaging assays like BRET appear promising in the current proteomic developments. So far, the major hurdle with BRET strategy was our inability to visualize the interactions of endogenous proteins. However, this is no longer an impediment, as Audet et al. have successfully reported the measurement of BRET signals in cell lines obtained from transgenic mice that are made to express β2-adrenergic receptor fused to RLuc (β2AR-RLuc) and βarrestin-2 fused to a GFP (GFP2-βarr2) (39). Even though this development does not count for an actual detection of endogenous proteins, it is definitely a leap in that direction. With the inception of BRET-based quantum dots (QDs) conjugates (40, 41), *in vivo* imaging in small animal models has been simplified. These new generations of BRET probes follow a similar approach as the conventional BRET systems and act as BRET acceptors for RLuc donor. Some of these QDs can emit at wavelengths as high as 800 nm, enabling the visualization of dynamic PPIs from deep tissues of small, live animals with better resolution.

## Conclusion

Bioluminescence-based live cell assays are becoming increasingly attractive in biological applications as they are rapid, fairly sensitive, cost effective and easy to perform, some are even acquiescent to high-throughput systems and offer several advantages in comparison to other *in vitro* systems. BRET has been utilized for developing diverse live cell-based assays, many of which have now been adapted in small animal research for tracking specific protein functions, phosphorylation, and protease activation events as well as screening genetic and chemical modulators. By making this technology versatile, their scope for BRET-based molecular imaging of biological events from living cells and subjects will continue to expand.

## Conflict of Interest Statement

The authors declare that the research was conducted in the absence of any commercial or financial relationships that could be construed as a potential conflict of interest.
